# Characterisation of antibiotic resistance of *Salmonella* isolated from dog treats in Japan

**DOI:** 10.1017/S0950268819000153

**Published:** 2019-03-06

**Authors:** S. Yukawa, I. Uchida, Y. Tamura, S. Ohshima, T. Hasegawa

**Affiliations:** 1 Department of Comparative Animal Science, College of Life Science, Kurashiki University of Science and The Arts, Okayama, Japan; 2 Course of Advanced Clinical Medicine, Division of Veterinary Science, Graduate School of Life and Environmental Sciences, Osaka Prefecture University, Osaka, Japan; 3 Department of Pathobiology, School of Veterinary Medicine, Rakuno Gakuen University, Hokkaido, Japan; 4 Center for Veterinary Drug Development, Rakuno Gakuen University, Hokkaido, Japan

**Keywords:** 4,5,12:i:–, antimicrobial resistance, *bla*_TEM_, dog treats, *Salmonella*

## Abstract

Dog treats might be contaminated with *Salmonella*. In Canada and the USA, outbreaks of human salmonellosis related to exposure to animal-derived dog treats were reported. Consequently, surveillance data on *Salmonella* contamination of dog treats have been gathered in many countries, but not in Japan. In the current study, we investigated whether dog treats in Japan were contaminated with *Salmonella*. Overall, 303 dog treats (of which 255 were domestically produced) were randomly collected and the presence of *Salmonella* investigated. Seven samples were positive for *Salmonella enterica* subsp. *enterica*. Among these isolates, three were identified as serovar 4,5,12:i:–; two were serovar Rissen; and two were serovar Thompson. All serovar 4,5,12:i:– and Thompson isolates were resistant to one or more drugs. Two serovar Rissen isolates were fully susceptible to all tested antimicrobial agents. All *Salmonella* isolates were susceptible to cefotaxime, ciprofloxacin and nalidixic acid. The gene *bla*_TEM_ was detected in two serovar 4,5,12:i:– isolates. The *bla*_CTX−M_ and *bla*_CMY_ genes were not detected in any isolates. This study demonstrated that dog treats in Japan could constitute a potential source of dog and human *Salmonella* infections, including multidrug-resistant *Salmonella* isolates.

## Introduction

*Salmonella* spp. are Gram-negative bacilli from the family Enterobacteriaceae that are capable of colonizing the intestinal tract of most vertebrates. Non-typhoidal *Salmonella* are important food-borne pathogens that cause gastroenteritis, bacteraemia and focal infections in human and animals [1]. Transmission of salmonellae to human typically occurs by ingesting meat, dairy products and other food contaminated by animal faeces, or by cross-contamination from food contaminated with salmonellae. Zoonotic transmission of non-typhoidal *Salmonella* can also occur by direct exposure to the faeces of reptiles, pets and other animals [2–6].

Several outbreaks of salmonellosis in human related to exposure to contaminated dog food and dog treat products have been reported. In 1999, laboratory and epidemiological investigations identified pig ear-based dog treats as a source of *Salmonella enterica* subsp. *enterica* serovar Infantis infection in human in Canada [7]. As a consequence of the Canadian outbreak, the Food and Drug Administration Center for Veterinary Medicine (FDA CVM) in the USA performed a retail sampling study investigating the prevalence of *Salmonella* in pet treats available in the US pet stores. Therein, 158 pet treats were collected, of which 41% were contaminated with *Salmonella* [8]. Twenty-four serotypes were identified, including *S.* Anatum, *S.* Typhimurium and *S.* Infantis. Of these, 36% were resistant to at least one antimicrobial, whereas 13% were resistant to four or more antimicrobials [8]. In 2002, 2004, 2005 and 2013, some human infections of *Salmonella* were attributed to pet treats in Canada and the USA [9–11]. A human infection in Canada in 2002 was caused by CMY-2 AmpC *β*-lactamase-producing *S.* Newport strains [9]. In many countries, the incidence of human infections caused by extended-spectrum cephalosporin-resistant *Salmonella* has increased dramatically [12–18]. In Japan, extended-spectrum cephalosporin-resistant *Salmonella* harbouring AmpC or extended-spectrum *β*-lactamase (ESBL) genes, such as *bla*_CTX−M−14_ and *bla*_CTX−M−15_, have been isolated from human [19, 20].

As no routine surveillance of dog treats for *Salmonella* contamination is performed in Japan, the main objective of the current study was to determine the current prevalence of *Salmonella* contamination in such dog treats. The second objective was to investigate the prevalence of *β*-lactam resistance among *Salmonella* from dog treats in Japan using a molecular approach to detect ESBL and AmpC *β*-lactamase genes.

## Methods

### *Salmonella* isolation and identification

A total of 303 product samples were collected, consisting of domestic products (*n* = 255) and imported products (*n* = 48). It was estimated that 300 samples would provide a 95% probability that at least one sample would be positive for *Salmonella*, assuming a minimum prevalence of 1%. All samples were collected in the Okayama and Osaka Prefectures in Japan from April 2016 to December 2016. The main pet supply chain from which we obtained our samples carried a variety of brands of imported and domestically packaged products. Other miscellaneous brands sold in supermarkets and in a chain store selling a large variety of products were purchased off the shelves. Prior to each sampling day, three stores were selected randomly in the scheduled city. We purchased 10 samples randomly at one store. We purchased three samples at another store, as only three were available. Other stores sold more than 30 kinds of dog treats. The samples were transported to the laboratory and kept at ambient temperature until analysis. *Salmonella* were isolated following the procedure of the US FDA Bacteriological Analytical Manual [21]. The isolates were identified using API 20E identification kits (bioMerieux, l'Etoile, France) and were serotyped by using slide and tube agglutination tests with commercially available antisera (Denka Seiken Co., Ltd., Tokyo, Japan). In addition, polymerase chain reaction (PCR) was used to serotype the *Salmonella* isolates [22].

### Antimicrobial susceptibility testing

*Escherichia coli* ATCC 25922 was used as the quality-control strain in the experiments. The minimum inhibitory concentrations (MICs) of the following drugs were determined using the microbroth dilution method on Eiken dry plates (Eiken Kagaku Co., Ltd., Tokyo, Japan): ampicillin (ABPC), cefazolin (CEZ), cefotaxime (CTX), chloramphenicol (CP), tetracycline (TC), gentamycin (GM), kanamycin (KM), nalidixic acid (NA), ciprofloxacin (CPFX) and trimethoprim (TMP). MIC breakpoints were interpreted according to the Clinical and Laboratory Standards Institute guidelines [23]. Susceptibility to streptomycin (SM) was determined by using the standard disk diffusion method [23] with Sensi-Discs (Japan Becton Dickinson Company, Tokyo, Japan). Isolates resistant to CTX were also tested for ESBL production, using a phenotypic confirmatory test [23].

### Detection of antimicrobial resistance genes

All DNA templates for analysis were prepared by the boiling method, as described elsewhere [24]. Briefly, bacterial cells were suspended in 200 µl of distilled water and boiled for 10 min. The cells were then pelleted by centrifugation for 1 min. The supernatants (5 µl) were used for PCR to detect the presence of the class 1 and class 2 integron genes and antimicrobial resistance genes. PCR was performed in a final volume of 25 µl using GoTaq^®^ Green master mix 2× (Promega, Madison, WI, USA), according to the manufacturer's instructions; primers are listed in Table 1.

**Table 1.** Primer sequences and expected PCR product sizes

Antimicrobial family	Resistance gene	Forward PCR primer (5′–3′)	Reverse PCR primer (5′–3′)	Product size (bp)	Ref.
*β*-lactams	*bla*_TEM_	ATCAGCAATAAACCAGC	CCCCGAAGAACGTTTTC	516	[25]
	*bla*_SHV_	AGGATTGACTGCCTTTTTG	ATTTGCTGATTTCGCTCG	392	[25]
	*bla*_OXA_	ATATCTCTACTGTTGCATCTCC	AAACCCTTCAAACCATCC	619	[25]
	*bla*_CTX−M_	CGCTTTGCGATGTGCAG	ACCGCGATATCGTTGGT	550	[26]
	*bla*_CMY_	GACAGCCTCTTTCTCCACA	TGGAACGAAGGCTACGTA	1007	[26]
	*bla*_CMY2_	CGATCCGGTCACGAAATACT	CCAGCCTAATCCCTGGTACA	556	[27]
Aminoglycosides	*aadA1*	CTCCGCAGTGGATGGCGG	GATCTGCGCGCGAGGCCA	631	[28]
	*aadA2*	CATTGAGCGCCATCTGGAAT	ACATTTCGCTCATCGCCGGC	500	[28]
TC	*tetA*	GCTGTCGGATCGTTTCGG	CATTCCGAGCATGAGTGCC	658	[28]
	*tetB*	CTGTCGCGGCATCGGTCAT	CAGGTAAAGCGATCCCACC	615	[28]
CP	*floR*	AATCACGGGCCACGCTGTATC	CGCCGTCATTCTTCACCTTC	215	[29]
	*cmlA*	CCGCCACGGTGTTGTTGTTATC	CACCTTGCCTGCCCATCATTAG	698	[30]
	*catA1*	AGTTGCTCAATGTACCTATAACC	TTGTAATTCATTAAGCATTCTGCC	547	[31]
TMP	*dfrA1*	CAATGGCTGTTGGTTGGAC	CCGGCTCGATGTCTATTGT	254	[28]
	*dfrA12*	TTCGCAGACTCACTGAGGG	CGGTTGAGACAAGCTCGAAT	330	[28]
Class 1 integron	*intI1*	GGGTCAAGGATCTGGATTTCG	ACATGGGTGTAAATCATCGTC	483	[32]
Class 2 integron	*intI2*	CACGGATATGCGACAAAAAGGT	GTAGCAAACGAGTGACGAAATG	788	[32]

TC, tetracycline; CP, chloramphenicol; TMP, trimethoprim

The resistant isolates were screened for the presence of 15 resistance genes corresponding to their resistance phenotypes. *β*-lactam antibiotic-resistant isolates (*n* = 3) were screened for the presence of the six *β*-lactamase genes (*bla*_TEM_, *bla*_SHV_, *bla*_OXA_, *bla*_CTX−M_, *bla*_CMY_ and *bla*_CMY2_) [25–27]. Aminoglycoside-resistant isolates (*n* = 3) were screened for the presence of the *aadA1* and *aadA2* genes [28]. TC-resistant isolates (*n* = 3) were screened for the presence of the *tetA* and *tetB* genes [28]. CP-resistant isolates (*n* = 2) were screened for the presence of the *floR*, *cmlA* and *catA1* genes [29–31]. TMP-resistant isolates (*n* = 4) were screened for the presence of the *dfrA1* and *dfrA12* genes [28]. All resistant isolates (*n* = 7) were screened for the presence of the *intI1* and *intI2* genes [32].

### Statistical methods

For all prevalence estimates, we calculated 95% confidence intervals using the Wilson score interval method [33]. Fisher's exact test was used to calculate the statistical differences between the prevalence of *Salmonella* in the imported and domestic dog treat samples.

## Results

### Isolation of *Salmonella* and identification of isolate serotypes

*S. enterica* subspecies *enterica* was isolated from seven (2.3%; 95% CI 1.1–4.7) of 303 dog treat samples, including five (2.0%; 95% CI 0.9–4.6) domestic and two (4.2%; 95% CI 1.2–14.0) imported products. There was no significant difference in the contamination levels between domestic and imported products. Three of these isolates represented serovar 4,5,12:i:–, two were serovar Rissen, and two were serovar Thompson. Five isolates were found in the domestic dog treats made from chicken, pig's ear and cow (Table 2). Two isolates were found in dog treats imported from Korea, made from pig's ear (Table 3).

**Table 2.** *Salmonella* isolated from domestic dog treats

Animal material	Number of samples	Number of samples positive for *Salmonella*	*S. enterica* subsp. *enterica* serotypes (number of isolates)
Chicken	67	1	Rissen (1)
Pig's ear	50	1	4,5,12:i:– (1)
Pig	6	0	
Cow	47	3	4,5,12:i:– (1); Thompson (2)
Deer	15	0	
Horse	7	0	
Turkey	5	0	
Boar	2	0	
Sheep	1	0	
Miscellaneous	55	0	
Total	255	5

**Table 3.** *Salmonella* isolated from imported dog treats

Country of origin	Animal material	Number of samples	Number of samples positive for *Salmonella*	*S. enterica* subs. *enterica* serotypes (number of isolates)
Korea	Pig's ear	15	2	Rissen (1); 4,5,12:i: – (1)
	Pig	1	0	
China	Chicken	12	0	
	Cow	2	0	
	Miscellaneous	5	0	
Vietnam	Miscellaneous	1	0	
New Zealand	Sheep	3	0	
	Cow	1	0	
	Deer	1	0	
	Miscellaneous	1	0	
USA	Turkey	2	0	
Unknown	Pig's ear	2	0	
	Chicken	2	0	
Total		48	2	

### Antimicrobial susceptibility profiles of the isolates

All *Salmonella* isolates were susceptible to CTX, CPFX and NA. Two isolates (serovar Rissen) (29% of all isolates) was fully susceptible to all tested antimicrobial agents (Tables 4 and 5). Five isolates were resistant to one or more drugs, including *Salmonella* serovars 4,5,12:i:– (*n* = 3) and Thompson (*n* = 2). Three isolates representing serovar 4,5,12:i:– were resistant to four and more antimicrobials. Because all *Salmonella* isolates were susceptible to CTX, ESBL production was not pursued.

**Table 4.** MICs for *Salmonella* isolates

Animal material	Country of origin	Serotype	MIC range (μg/ml)									
			ABPC (1–128)	CEZ (1–128)	CTX (0.5–64)	GM (0.8–64)	KM (1–128)	TC (0.5–64)	NA (1–128)	CPFX (0.03–4)	CP (1–128)	TMP (0.25–16)
Pig's ear	Korea	Rissen	2	2	⩽0.5	2	8	4	8	0.06	8	0.5
Pig's ear	Korea	4,5,2:i:–	>128	16	⩽0.5	2	8	>64	8	0.06	8	⩽0.25
Pig's ear	Japan	4,5,12:i:–	>128	16	⩽0.5	2	>128	>64	8	0.12	>128	>16
Chicken	Japan	Rissen	4	⩽1	⩽0.5	1	8	4	8	0.06	8	0.5
Cow	Japan	4,5,12:i:–	>128	2	⩽0.5	0.5	2	>64	8	⩽0.03	>128	>16
Cow	Japan	Thompson	⩽1	⩽1	⩽0.5	1	4	2	8	⩽0.03	4	>16
Cow	Japan	Thompson	⩽1	⩽1	⩽0.5	⩽0.5	2	2	8	⩽0.03	4	>16

ABPC, ampicillin; CEZ, cefazolin; CTX, cefotaxime; GM, gentamycin; KM, kanamycin; TC, tetracycline; NA, nalidixic acid; CPFX, ciprofloxacin; CP, chloramphenicol; TMP, trimethoprim.

**Table 5.** Summary of the *Salmonella* isolate resistance profiles

Animal material	Country of origin	Serotype	Resistance phenotypes	Resistance genes
Pig's ear	Korea	Rissen		
Pig's ear	Korea	4,5,12:i:–	ABPC, CEZ, SM, TC	*bla*_TEM_, *tetB*
Pig's ear	Japan	4,5,12:i:–	ABPC, CEZ, SM, KM, TC, CP, TMP	*intI1*, *tetA*, *tetB*, *aadA1*, *aadA2*, *floR*, *catA1*, *dfrA12*
Chicken	Japan	Rissen		
Cow	Japan	4,5,12:i:–	ABPC, SM, TC, CP, TMP	*intI1*, *bla*_TEM_, *aadA1*, *aadA2*, *tetB*, *floR*, *catA1*, *cmlA*, *dfrA12*
Cow	Japan	Thompson	TMP	
Cow	Japan	Thompson	TMP	

ABPC, ampicillin; CEZ, cefazolin; SM, streptomycin; KM, kanamycin; TC, tetracycline; CP, chloramphenicol; TMP, trimethoprim

### Detection of antimicrobial resistance genes harboured by the isolates

PCR screening of the *Salmonella* isolates for the presence of integron genes revealed that two isolates were positive for the class 1 integron gene and none were positive for the class 2 integron gene (Table 5). The distribution of the various resistance genes in the isolates is shown in Table 5. Two of the three ABPC-resistant isolates contained the *bla*_TEM_ gene; none of the ABPC-resistant isolates contained any other *β*-lactamase genes. The *tetB* gene was detected in all (three) TC-resistant isolates. The *floR* and *catA1* genes were detected in all (two) CP-resistant isolates. The *aadA1* and *aadA2* genes were detected in two out of the three SM-resistant isolates. The *dfrA12* gene was detected in two out of the four TMP-resistant isolates.

## Discussion

In the current study, we showed that dog treats in Japan might harbour *Salmonella*. The determined prevalence of *Salmonella* in dog treats from Japan was 2%. Although the difference was not significant, the prevalence of *Salmonella* was slightly higher for imported treats than for domestic treats. A survey performed in the UK by Willis identified *Salmonella* in 7.8% imported dog chews [34]. In this aforementioned study, samples imported from Asia or South Africa were the main target of the survey. A total of 2369 dog chew samples imported from Thailand, China, India, Sri Lanka, Argentina, Brazil, Colombia and the USA were examined at public health laboratories in Ashford, Chelmsford, Ipswich, London and Southampton. Overall, *Salmonella* species were detected in 184 samples (7.8%). Wong *et al*. found *Salmonella* in 6.7% dog chews in New Zealand [35]. The authors collected 600 samples, consisting of New Zealand-produced (domestic) and imported samples. Most samples were purchased from two stores of a major pet supply chain in Christchurch and by direct mail order from a major supplier in Wellington, New Zealand; there was no significant difference in the contamination levels between imported and domestic samples. The prevalence of *Salmonella* identified in the current study was lower than that of previous reports. For instance, Clark *et al*. reported that 51% of retail Canadian pig ear treats collected from Alberta, Saskatchewan, Ontario, Québec, Newfoundland and Nova Scotia were positive for *Salmonella* [7]. White *et al*. found 41% of retail dog treats collected in the USA by 16 district offices and seven regional laboratories of the FDA to be positive for *Salmonella* in the USA [8]. Li *et al*. reported that the prevalence of *Salmonella* in pet food or pet treats collected under the FDA CVM Feed Contaminants Program in the years 2007–2009 was significantly (*P* < 0.05) lower than that reported for the years 2002–2006 [36]. It is thought that this reduction of *Salmonella* prevalence is associated with the various countermeasures undertaken by each country. The American Feed Industry Association [37] and the European Pet Food Industry Federation [38] have developed guidelines for the manufacturing of pet products. In addition, to monitor the trend of *Salmonella* contamination in animal feed, since 2002, the FDA CVM has established a *Salmonella* surveillance programme, which includes dog treats, in the USA. Subsequently, the FDA CVM has established a second surveillance programme, including dog treats, in the USA. In Japan, the Law for Ensuring the Safety of Pet Food came into force in 2009, and the Ministry of Agriculture, Forestry, and Fisheries published a proper manufacturing manual for pet food (including dog treats) in 2014. Nevertheless, the Japanese government have not carried out any *Salmonella* surveillance programmes that include dog treats. Since *Salmonella* contamination was indeed detected in dog treats in the current study, the government of Japan should take stronger measures to counteract the possible associated health threat. It is not known how many dog treats are sold in Japan. Neither is it known how many companies are manufacturing dog treats in Japan, as the government of Japan have not published a report with these data. However, the government of Canada have published a report about dog treats in Japan, which estimated that retail sales of dog treats and mixers would reach US$553.9 million in 2016 [39]. However, the figures described in this report were not examined by the government itself but were estimated by a market research company. Therefore, we could not verify the accuracy of the content. Thus, the government of Japan should make efforts to determine the distribution volume and sales of dog treats in Japan, which would help determine sample size in future studies of this nature.

In the current study, the *Salmonella*-positive dog treats originated from Japan and Korea. However, we also found dog treats of unknown country of origin. In Japan, the Law for Ensuring the Safety of Pet Food states that sellers of pet food, including dog treats, must label the content, country of origin, expiration date, materials, location, company name and company location on the pet food products.

An increasing incidence of multidrug-resistant *Salmonella* has been widely reported in the past and is presumably attributed to the extensive use of antimicrobial agents in human and veterinary medicine [40]. In a US study, White *et al*. found that *Salmonella* isolated from dog treats harboured class 1 integrons [8]. In Japan, Futagawa-Saito *et al*. reported that the rates of antimicrobial resistance among faecal isolates from healthy pigs obtained in the years 2004–2005 were significantly higher than those of isolates from the years 1998–1999 [41]. In the current study, we detected the *bla*_TEM_ gene but not the *bla*_CTX−M_, *bla*_CMY_ or *bla*_CMY2_ genes in *Salmonella* isolates from dog treats in Japan. More extensive monitoring of dog treats must be undertaken as part of surveillance of multidrug-resistant *Salmonella*.

In conclusion, a small percentage of dog treats in Japan are contaminated with *Salmonella*, including antimicrobial-resistant isolates. In the USA, an outbreak of *Salmonella* Typhimurium occurred in humans that had been exposed to dog treats [11]. Therefore, care should be taken when handling dog treats. It is recommended that people wash their hands after feeding dogs and after any contact with dog treats [42]. Veterinarians have the responsibility to disseminate accurate information about the potential contamination risks, so that appropriate precautions can be implemented. In Japan, there are currently no reports of human salmonellosis caused by dog treats. Although the risk of salmonellosis from contaminated dog treats may be low, adhering to safety recommendations will help to minimise the risk of infections with *Salmonella* in dogs and family members.
